# What dictates income in New York City? SHAP analysis of income estimation based on Socio-economic and Spatial Information Gaussian Processes (SSIG)

**DOI:** 10.1057/s41599-023-01548-7

**Published:** 2023-02-15

**Authors:** Ruiqiao Bai, Jacqueline C. K. Lam, Victor O. K. Li

**Affiliations:** Department of Electrical and Electronic Engineering, The University of Hong Kong, Hong Kong, China

**Keywords:** Economics, Social policy

## Abstract

Income inequality presents a key challenge to urban sustainability across the developed economies. Traditionally, accurate high granularity income data are generally obtained from field surveys. However, due to privacy considerations, field subjects are hesitant to provide accurate personal income data. A *Socio-economic & Spatial-Information-GP* (SSIG) model is thereby developed to estimate district-based high granularity income for New York City (NYC). As compared to the state-of-the-art Gaussian Processes (GP) income estimation model based entirely on spatial information, SSIG incorporates socio-economic domain-specific knowledge into a GP model. For SSIG to be explainable, SHapley Additive exPlanations (SHAP) analysis is undertaken to evaluate the relative contribution of various key individual socio-economic variables to district-based per-capita and median household income in NYC. Differentiating from traditional income inequality studies based predominantly on linear or log-linear regression model, SSIG presents a novel income-based model architecture, capable of modelling complex non-linear relationships. In parallel, SHAP analysis serves an effective analytical tool for identifying the key attributes to income inequality. Results have shown that SSIG surpasses other state-of-the-art baselines in estimation accuracy, as far as per-capita and median household income estimation at the Tract-level and the ZIP-level in NYC are concerned. SHAP results have indicated that having a bachelor or a postgraduate degree can accurately predict income in NYC, despite that between-district income inequality due to Sex/Race remains prevalent. SHAP has further confirmed that between-district income gap is more associated with Race than Sex. Furthermore, ablation study shows that socio-economic information is more predictive of income at the ZIP-level, relative to the spatial information. This study carries significant implications for policy-making in a developed context. To promote urban economic sustainability in NYC, policymakers can attend to the growing income disparity (income inequality) contributed by Sex and Race, while giving more higher education opportunities to residents in the lower-income districts, as the estimated per-capita income is more sensitive to the proportion of adults ≥25 holding a bachelor’s degree. Finally, interpretative SHAP analysis is useful for investigating the relative contribution of socio-economic inputs to any predicted outputs in future machine-learning-driven socio-economic analyses.

## Introduction

### Motivation and research objectives

Income inequality presents a key challenge to urban sustainability in the developed economies (Cantante, 2020; Chancel et al., 2018). Cities having big income divide may also be characterised by unequal access to higher education, employment, or safety protection opportunities (Schneider, 2016; Shutters et al., 2022; Wan et al., 2022). To address such inequality, accurate representation of income distribution in high granularity and its determinants are crucial for evidence-based policy-making (Suel et al., 2018). Given better welfare allocation, citizens in developed economies tend to spend more; a citizen’s income level can better reflect an individual’s concurrent spending on goods and services (Chen et al., 2010; Pfoertner et al., 2011). In addition, in contrast to the unilateral distribution of low-income households, as evidenced in some low-income economies, developed economies are more susceptible to a higher risk of intra-city income inequality, resulting in a higher demand for data transparency. More accurate and fine-grained income data at the intra-city level are important for facilitating income-related policy decision-makings. In some countries, fiscal policies have targeted to narrow the income gap across different socio-economic groups (Piotrowski and Van Ryzin, 2007; Suel et al., 2021; Tsui et al., 2018), thereby improving urban sustainability in the developed economies.

Traditionally, collecting accurate income data of a higher spatial granularity via field surveys (Gebru et al., 2017) is labour-intensive. These data can be social security statistics or administrative data (Fritzell et al., 2011). To avoid sensitivity due to disclosure of personal income information, field-survey participants might hesitate to provide accurate information (Davern et al., 2005; Kim et al., 2007). In developed countries such as the U.K., such information is not allowed to be disclosed in census reports (Suss, 2021).

To gather fine-grained income data for developed economies, two types of income estimation models can be used (for further details, see Literature Review). The first infers income from socio-economic variables collected via field surveys. The second relies on machine learning or big data collection methods other than field surveys. In an AI-driven income estimation study (Bai et al., 2020), three outstanding machine-learning-based high granularity income estimation models for developed economies had been developed, including, the *GP-Mixed-Siamese-like-Double-Ridge* model, the *Mixed-Siamese-like* model and the *Spatial-Information-GP* model. In particular, the *Spatial-Information-GP* model outperforms the other two in terms of model accuracy, and creates less data collection burden (Bai et al., 2020). This model took only the latitudes and the longitudes of district centroids as the inputs to the Gaussian Processes (GP) (Williams and Rasmussen, 2006).

Along the line of district-based income estimation, previous modelling studies explored the contribution of individual socio-economic variables on district-based income estimation across the developed economies (Almada, 2004; Fullerton Jr et al., 2014; Fullerton Jr et al., 2010; Fullerton, 2001; Morales, 2012), which presents limits (for further details, see Literature Review). Some key variables are yet to be incorporated into the models in these studies; the spatial resolution of these estimated income studies needs to be enhanced further; previous machine-learning models are yet to be able to fully capture the complex non-linear relationships between the socio-economic and the income variables.

One major purpose of studying the effects of socio-economic variables on district-based income distribution is to study in greater details any traces of income inequality. Analysing income inequality in urban areas is challenging given the many forces at play (Matthew and Brodersen, 2018). There are two types of district-based income inequality, namely, within-district inequality and between-district inequality. For large-scale field surveys such as the American Community Survey, data indicative of within-district inequality, such as Within-district Gini Index and Share of Aggregate Household Income by Quintile, are available. However, data indicative of between-district income inequality are missing (ACS, 2021). Hence, we will focus on between-district inequality given that only the 5-year average income data is available in the CR (ACS, 2021).

Built upon the previous study (Bai et al., 2020), this study attempts to answer two research questions: First, by incorporating the socio-economic data collected from the field surveys into the *Spatial-Information-GP* model, would the accuracy of income estimation across the developed economies be improved? Second, what socio-economic variables inputted to our machine-learning and big data-based models best contribute to income estimation in New York City (NYC)? To address these two questions, we propose a novel *Socio-economic and Spatial-Information-GP* (SSIG) model, incorporating ten important socio-economic variables (based on thorough literature review) into a *Spatial-Information-GP* model. Using the field socio-economic data collected for NYC, we compare the SSIG performance with that of other comparable state-of-the-art income estimation models in a developed context. We conduct the SHapley Additive exPlanations (SHAP) analysis (Lundberg and Lee, 2017) to understand the effects of individual socio-economic variables on income estimation. Our SSIG model presents a novel district-based income estimation architecture, capable of modelling the complex non-linear relationships between income and non-income variables. SSIG differs from traditional income inequality-based modelling, which was based on linear or log-linear regression. SSIG also differs significantly from previous study (Bai et al., 2020), which estimated income without taking into account the socio-economic data from field surveys.

### Literature review

Two types of income estimation models have been adopted to estimate fine-grained income data in developed economies. The first one mainly inferred incomes from the socio-economic variables collected via field surveys. Supplementary Table S1 summarises the socio-economic variables adopted in traditional models of high granularity income estimation across developed economies (Almada, 2004; Dodge, 2003; Fullerton Jr et al., 2014; Fullerton Jr et al., 2010; Fullerton, 2001; Morales, 2012). Multiple socio-economic variables, such as education level and employment, were incorporated into these models. The second type mainly relied on machine-learning and big data-driven estimation models instead of field surveys. Some of these models used house price as a proxy (Määttänen and Terviö, 2014; Piggott, 2015). Although house price data can also be gathered via field surveys (Määttänen and Terviö, 2014), due to preference for data collection via electronic records, most inclined to collect information online, either via commercial websites or official land registries (Piggott, 2015). In addition, multiple data types, such as night-time/day-time satellite images or street views (Abitbol and Karsai, 2020; Acharya et al., 2017; Gebru et al., 2017; Glaeser et al., 2018; Mellander et al., 2015; Suel et al., 2021; Suel et al., 2019), district-based spatial information (e.g., the latitude and the longitude) (Suel et al., 2018), human mobility records (Smith et al., 2013), restaurant information (Block et al., 2004), and socio-media records (Hristova et al., 2016), were utilised in fine-grained district-based income estimation. Along the line of machine-learning and big data-driven models, Bai et al. (2020) previously developed three fine-grained income estimation models for the developed economies, including the *GP-Mixed-Siamese-like-Double-Ridge* model, the *Mixed-Siamese-like* model, and the *Spatial-Information-GP* model, with inputs from non-field-survey big data only.

With respect to district-based income estimation, former income estimation models explored the effects of socio-economic variables on estimated income distribution across the developed economies (Almada, 2004; Fullerton Jr et al., 2014; Fullerton Jr et al., 2010; Fullerton, 2001; Morales, 2012). Among the socio-economic variables investigated in these studies, educational attainment, as represented by the proportion of adults ≥25 holding a bachelor degree or above (Fullerton Jr et al., 2014; Fullerton Jr et al., 2010; Morales, 2012), was a good predictor of one’s income level. In general, a district with an elevated the proportion of highly educated residents tended to have a higher income (Dodge, 2003; Fullerton Jr et al., 2014; Fullerton Jr et al., 2010; Morales, 2012), likely attributable to enhanced productivity due to higher educational attainment (Jones, 2001). Other socio-economic variables such as employment (Almada, 2004; Dodge, 2003; Fullerton Jr et al., 2014), age (Almada, 2004; Fullerton Jr et al., 2014; Fullerton, 2001; Morales, 2012) and population density (Almada, 2004; Fullerton Jr et al., 2014; Fullerton Jr et al., 2010; Fullerton, 2001; Morales, 2012), were also reported to associate with district-based income, since these variables potentially influenced district-based productivity. However, relying on these socio-economic variables alone for fine-grained income estimation presents limits. Though race and sex were mostly taken as having a high correlation with income (Akee et al., 2019; Hinze, 2000), most of these income-driven studies had focused on a limited set of socio-economic variables in limited sample size, instead of taking all relevant socio-economic variables as the input features for fine-grained income estimation by more sophisticated machine-learning models. Besides, these field-survey-based modelling mainly focused on low-resolution county-level instead of high-resolution district-based income estimation (Almada, 2004; Dodge, 2003; Fullerton Jr et al., 2014; Fullerton Jr et al., 2010; Fullerton, 2001; Morales, 2012). Such income data were of insufficient resolution to capture intra-city income distribution. Furthermore, these traditional income estimation models mostly relied on simple machine-learning techniques, such as linear regression (Almada, 2004; Fullerton, 2001) and log-linear regression (Fullerton Jr et al., 2014; Fullerton Jr et al., 2010; Morales, 2012). These simple machine-learning models (Maulud and Abdulazeez, 2020) also could not sufficiently capture the complex non-linear relationship between socio-economic status and income in high spatial resolution.

## Methods

### Data collection and pre-processing

#### Labelled data

Our study focusses on NYC, one of the most advanced global economies. In addition, Table 1 presents the Between-district Gini Coefficients (StatisticalHelp, 2022) and Decile Dispersion Ratios (the ratio between the average income of the richest 10% and the poorest 10% districts) (WBG, 2022) for different types of income at different granularities. These values indicate the existence of between-district income inequality in NYC, deserving further investigation. District income in NYC was obtained from the 2015–019 American Community Survey (a 5-year estimate), corresponding to the average income across the 5-year period (ACS, 2021). American Community Survey collects data based on a 1/40 housing units ratio annually (ESRI, 2021). Owing to high labour intensity, smaller districts (i.e., districts with <65,000 residents) are interviewed less frequently than larger districts (Gebru et al., 2017). The credibility of such analyses is heavily constrained by their restricted sample size. Currently, there is no yearly fine-grained income data in NYC, which gives a finer data granularity as compared to the 5-year average. In future, yearly data are preferred to 5-year aggregate data, as this makes possible more accurate analyses of between-district income inequality. Two district-based average income variables were used as labels: per-capita income and median household income in NYC (Table S2 presents the data source and the corresponding time frame). Average income across two geographical levels, including the Tract-level and the ZIP-level, were used as labelled data, collected from Census Reporter (CR, 2021). Finally, per-capita income data across 2117 Tract-level districts and 180 ZIP-level districts, together with household income data across 2095 Tract-level and 179 ZIP-level districts, had been identified from the 5-year census and incorporated into our model.

**Table 1:** Between-district income inequality variables.

Between-district income inequality variable	Tract-level		ZIP-level	
	Per-capita income	Median household income	Per-capita income	Median household income
Gini coefficient	0.33	0.26	0.35	0.26
Decile dispersion ratio	7.87	6.05	7.73	5.59

Note: This table presents between-district income inequality variables calculated based on per-capita income and median household income at the Tract-level and the ZIP-level in NYC.

#### Input data

**Fig. 1:**
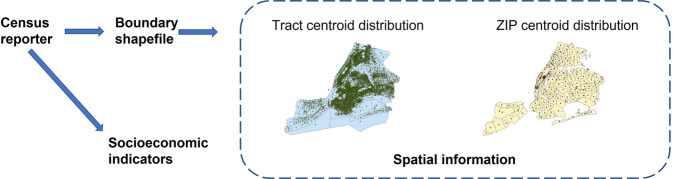
Input data used in SSIG.

As shown in Fig. 1, two types of inputs were used in our SSIG model: the spatial information of individual districts, and the socio-economic variables collected via the field survey, the Census Reporter (Table S2 presents the data source and the corresponding time frame).

Spatial information covers both the latitude and the longitude of any district centroid. The information was derived from the district boundary shapefile offered by Census Reporter (CR, 2021).

As for socio-economic variables collected via the field survey, based on the previous models that estimated fine-grained district-based income in the developed economies (Almada, 2004; Dodge, 2003; Fullerton Jr et al., 2014; Fullerton Jr et al., 2010; Fullerton, 2001; Morales, 2012), and given data availability at the Tract-level and the ZIP-level in NYC (collected by 2015–2019 American Community Survey) (ACS, 2021; CR, 2021), ten socio-economic variables were selected carefully via thorough literature review as the inputs to our SSIG model (see Table 2). Specifically, two variables were used to represent educational attainment, including the proportion of adults aged 25 or above holding a bachelor degree (≥25UDG) and the proportion of adults aged 25 or above holding a postgraduate degree (≥25PGD). One variable, the unemployment rate (Un-employ), was used to represent the employment status. As for the age structure of the population, the proportion of population aged 65 or above (≥65) and the proportion of population who are younger than 18 (<18) were incorporated into our model. Besides, the population density (Pop-density), calculated by the total population of each district divided by the corresponding area size, with the information provided by the shapefiles provided) (CR, 2021), is used as an input to our SSIG model. The sex structure was represented by the proportion of Males in the total population of a district. Finally, the proportion of Black or African American population, the Asian population and the White population (Black or African, Asian and White) were used to reflect the racial distribution of individual districts. All proportion values were taken as zeros if the denominators (total population) reported for the corresponding districts were taken as zeros. For NYC districts that did not have income data, their corresponding income values were excluded from model training and validation.

**Table 2:** Detailed descriptions of the socio-economic features of the SSIG model.

Name	Description
≥25UDG	The proportion of adults ≥25 holding a bachelor degree
≥25PGD	The proportion of adults ≥25 holding a postgraduate degree
Un-employ	Unemployment rate
≥65	The proportion of the population who are 65 or above
<18	The proportion of the population who are younger than 18
Pop-density	Population density
Male	The proportion of Male persons
Black or African	The proportion of the Black or African American population
Asian	The proportion of the Asian population
White	The proportion of the White population

Note: This table describes the corresponding socio-economic input features of the SSIG model.

Besides, for model comparison, we incorporated new data types for baseline models, including the house price, the day-time satellite image, and the street view (Table S2 presents data source and corresponding time frame). The house price information in 2019 was obtained from NYC Department of Finance (NYCDF, 2021). Each house price datapoint represented a real housing transaction; the latitude and the longitude of each building were identified from the official map searching tool (NYCGOV, 2021b). Day-time satellite images, captured in 2018, were gathered from NYC Government (NYCGOV, 2021a). Street view images taken from 2018 to 2020 were obtained from Google Street View Static API (Google, 2021). Data processing and cleaning methodologies adopted for this study are consistent with our previous study (Bai et al., 2020).

### Model development

One previous study suggested that the *Spatial-Information-GP* model can achieve high-income estimation accuracy with minimal burdens of data collection (Bai et al., 2020). Specifically, the model took the latitudes and the longitudes of district centroids as the inputs of a GP model (Williams and Rasmussen, 2006).

The GP model is a non-linear model developed based on a Bayesian approach, carrying a Gaussian prior over the parameters (Williams and Rasmussen, 2006). Specifically, it can be expressed by Eq. (1)(Williams and Rasmussen, 2006):

1 $$f\left( x \right) = \varphi \left( x \right)^Tw$$

where *x* is the input feature vector, *w* is the parameter vector that follows a Gaussian distribution *N*(*μ*, ∑), *φ*(.) is a function that maps the input vector to a high-dimensional space and determined by a kernel function *k*(*x*, *x*′) = *φ*(*x*)^*Т*^∑*φ*(*x*′). The kernel function defines the covariance between each pair of inputs *x* and *x*′ (Williams and Rasmussen, 2006). The Matern-3/2 kernel was adopted for this study (Bai et al., 2020), which can be defined by Eq. (2) (GPy, 2012; Williams and Rasmussen, 2006):

2 $$k\left( {x,x^\prime } \right) = \left( {1 + \frac{{\sqrt 3 \left| {x - x^\prime } \right|}}{l}} \right)\exp \left( { - \frac{{\sqrt 3 \left| {x - x^\prime } \right|}}{l}} \right)$$

where *l* is a hyperparameter (*l* = 1).

In this machine-learning big data-driven SSIG study, we further took both socio-economic variables and spatial information as the inputs to a GP model.

We also compared the model performance with five state-of-the-art models with relatively high validation accuracy, including the *GP-Mixed-Siamese-like-Double-Ridge* model, the *Mixed-Siamese-like-GP* model, the *Mixed-Siamese-like-Random-Forest* model, the *Mixed-Spatial-Siamese-like* model, and the *Mixed-Siamese-like* model (Bai et al., 2020). The architectures of those models were detailed in Bai et al. (2020).

### Ablation study

To conduct the ablation study, different groups of input features were fed into the GP model. Specifically, we compared our model (SSIG) with two GP models that rely entirely on the socio-economic data only (*Socio-economic-Information-GP* model), and entirely on spatial information only (*Spatial-Information-GP* model).

### SHAP analysis

To measure the contribution/impact of each socio-economic variable in income estimation, SHAP values of the socio-economic variables in SSIG were calculated (Lundberg and Lee, 2017). SHAP values were developed based on the co-operative game theory (Lipovetsky and Conklin, 2001). It took regression as a means of establishing coalitions among different players (i.e., input variables) to maximise the total score of these players (i.e., how fitting is the regression) (Lipovetsky and Conklin, 2001). Various combinations of players were taken to form coalitions, while each SHAP value measures the average contribution of each player across all possible combinations (Lipovetsky and Conklin, 2001). The SHAP value of individual input feature *j* of the model *f*, denoted by *ϕ*^*j*^ *(f)*, is defined as Eq. (3) (Lundberg and Lee, 2017):

3 $$\phi ^j\left( f \right) = \frac{1}{{\left| N \right|!}}\mathop {\sum}\nolimits_{S \subseteq N\backslash \left\{ j \right\}} {\left| S \right|!\left( {\left| N \right| - \left| S \right| - 1} \right)!\left[ {f\left( {S \cup \left\{ j \right\}} \right) - f\left( S \right)} \right]}$$

where *N* represents the set of all features and *S* denotes a subset of features (Lundberg and Lee, 2017). *f*(*S*) is defined as *E*(*f*(*x*)|*x*_*s*_), where *E*(.) is the expectation function, *x* is a set of values for all input features, and *x*_*s*_ is a set of values for input features in *S* (Lundberg and Lee, 2017). Hence, the SHAP value of a feature represents the weighted average of the feature’s expected impact across all possible feature combinations. Owing to the additivity of the SHAP value, the SHAP value of multiple features is calculated by summing up the SHAP values of all individual features (Lundberg and Lee, 2017).

Our study based on SHAP analysis presents several advantages, when compared to traditional studies that analysed contributions of input variables based on parameters of linear or log-linear regressions (Almada, 2004; Fullerton Jr et al., 2014; Fullerton Jr et al., 2010; Fullerton, 2001; Morales, 2012). A distinctive advantage of SHAP being that, instead of being restricted by linear or log-linear assumptions, it can freely adapt to various complex non-linear machine-learning models, which may result in higher accuracy. In addition, it has been proven empirically that SHAP can provide consistent results in the presence of multi-collinearity (Lipovetsky and Conklin, 2001). In reality, multi-collinearity can lead to high variance across estimated parameters in some traditional statistical models, due to randomness in sampling, subsequently reducing the credibility of estimated contributions of input variables (Lipovetsky and Conklin, 2001).

**Fig. 2:**
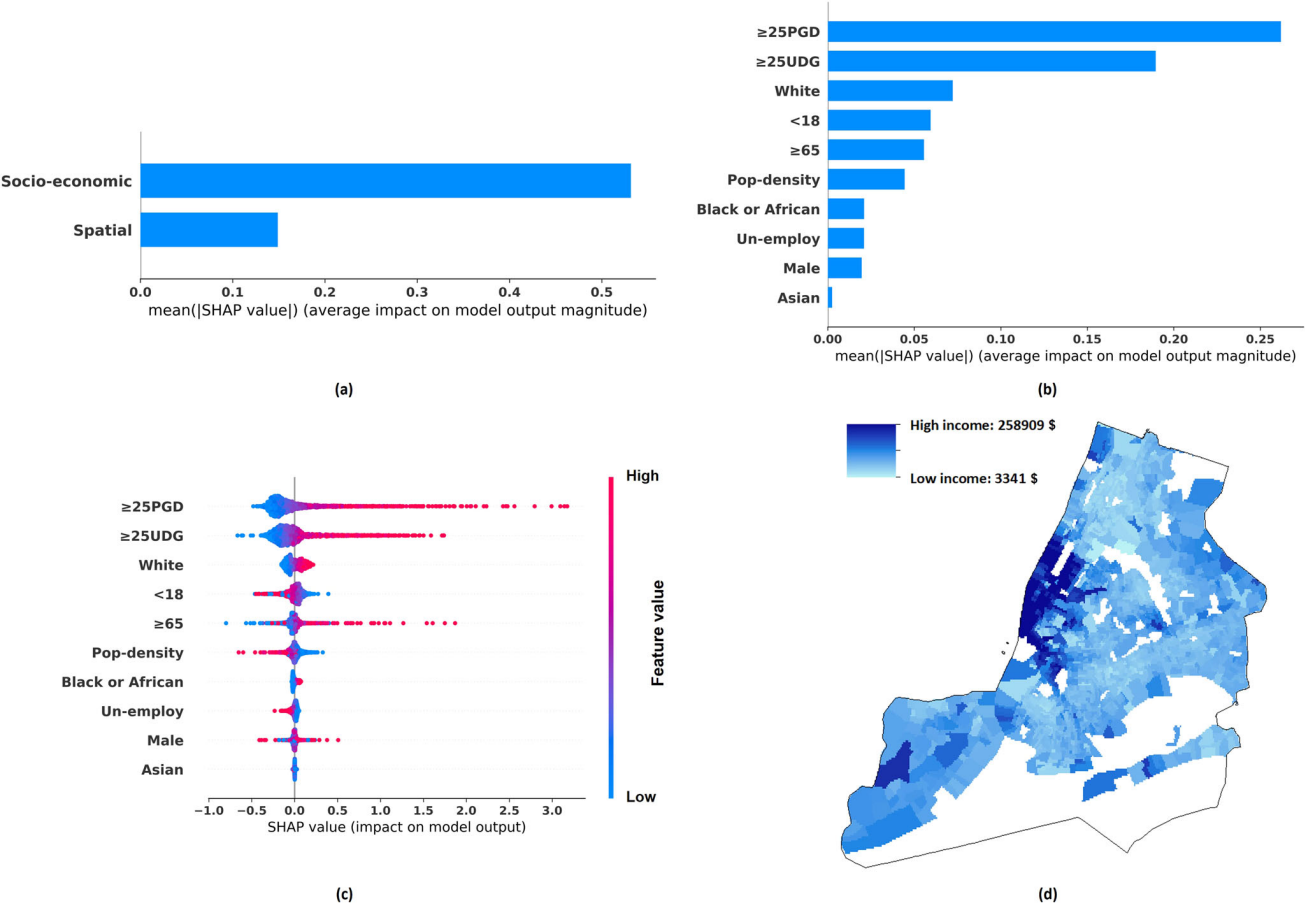
Tract-level per-capita income distribution and SHAP analysis results. **a** The mean absolute SHAP value indicating the total contribution of spatial or socio-economic features to Tract-level per-capita income estimation. **b** The mean absolute SHAP value indicating the contribution of individual socio-economic features to Tract-level per-capita income estimation. **c** The SHAP value indicating the contribution of individual socio-economic features to Tract-level per-capita income estimation, with each dot corresponding to a value of a particular Tract/district. **d** Tract-level per-capita income distribution.

Specifically, SHAP figures can be used to study the key socio-economic variables associated with the income gap among different districts. The first one is a bar chart (see Fig. 2(a), (b)), which presents the mean absolute value of an individual variable or a group variable’s SHAP value. The longer the bar, the higher the effect of an individual variable on the estimated income. The second one is a scatter plot (see Fig. 2(c) as an example). Specifically, Fig. 2(c) shows the SHAP value distribution of a socio-economic variable, with each dot representing the value of a district in NYC. Each dot is coloured according to the variable’s value in a particular district, with blue representing a lower value and red representing a higher value. If the variable is associated with a decrement in the estimated income, the dot will be shown on the left side of the figure, indicating that variable has a negative SHAP value (and vice versa). Hence, a scatter plot can be used to check if a socio-economic variable of a higher value can lead to higher/lower district-based income, thus showing which factors are most significant in shaping between-district income inequality.

## Results

### Model performance

SSIG was implemented by the GPy package (GPy, 2012). Labels and input features were normalised (subtracting the mean and dividing by the standard deviation) before the training. Four types of evaluation matrix were used to compare model performance, including the square of the Pearson correlation coefficient (*r*^2^), Coefficient of Determination (CoD), Root Mean Square Error (RMSE), and Mean Absolute Error (MAE) (Jean et al., 2016; Perez et al., 2019).

Table 3 presents the results of the fivefold validation for per-capita income and median household income estimation at the Tract and the ZIP-level in NYC. After incorporating the socio-economic variables into the SSIG model, as compared to other state-of-the-art baseline models, the income estimation accuracy of the fivefold validation has been improved further. It must be noted that only the NYC districts with available ground truth income data were incorporated in the fivefold validation (NYC districts where income data are available also have socio-economic variable data available). Besides, some baseline models, including the *GP-Mixed-Siamese-like-Double-Ridge* model, the *Mixed-Siamese-like-GP* model, the *Mixed-Siamese-like-Random-Forest* model, the *Mixed-Spatial-Siamese-like* model, and the *Mixed-Siamese-like* model (Bai et al., 2020), have only covered those districts where street view imaging data were available.

**Table 3:** Results of a fivefold validation on SSIG and other baselines.

Model	Tract-level								ZIP-level							
	Per-capita income				Median household income				Per-capita income				Median household income			
	*r*^2^	CoD	RMSE	MAE	*r*^2^	CoD	RMSE	MAE	*r*^2^	CoD	RMSE	MAE	*r*^2^	CoD	RMSE	MAE
SSIG	**0.89**	0.89	9619	5424	**0.85**	0.84	13,655	10,040	**0.94**	0.91	9475	5418	**0.85**	0.84	14,654	10,036
Spatial information GP	0.84	0.84	11,414	6865	0.69	0.68	19,507	13,466	0.87	0.83	12,571	8509	0.75	0.72	19,174	13,839
Socio-economic information GP	0.84	0.84	11,604	6217	0.81	0.80	15,317	11,351	0.94	0.91	9091	5416	0.85	0.82	15,035	10,422
Mixed Siamese-like Double Ridge	0.85	0.84	11,314	7014	0.73	0.73	18,146	12,957	0.88	0.86	11,619	7850	0.77	0.75	18,070	13,182
Mixed Siamese-like GP	0.81	0.80	12,866	7722	0.69	0.69	19,437	13,933	0.85	0.83	12,994	8885	0.74	0.71	19,690	14,326
Mixed Siamese-like Random Forest	0.76	0.75	14,427	9003	0.61	0.60	21,857	15,602	0.83	0.80	13,566	9252	0.76	0.73	19,147	13,652
Mixed Spatial Siamese-like	0.77	0.76	14,043	9112	0.63	0.63	21,032	15,214	0.85	0.82	13,199	8819	0.72	0.69	19,961	14,017
Mixed Siamese-like	0.77	0.77	13,882	9109	0.66	0.65	20,398	14,999	0.86	0.84	12,723	8725	0.75	0.72	19,578	14,112

Note: This table presents the results of a fivefold validation for per-capita income and median household income estimation at the Tract and the ZIP-level in NYC.

### Ablation study

The results of the ablation study, including the fivefold validation of the SSIG model, the *Socio-economic-Information-GP* model and the *Spatial-Information-GP* model are shown in Table 4. It can be found that, in most cases, the SSIG model tends to outperform the GP model, which relies only on the socio-economic variables as the inputs, and the GP model, which relies only on the spatial information as the input.

**Table 4:** Results of ablation study (a fivefold validation).

Model	Tract-level								ZIP-level							
	Per-capita income				Median household income				Per-capita income				Median household income			
	*r*^2^	CoD	RMSE	MAE	*r*^2^	CoD	RMSE	MAE	*r*^2^	CoD	RMSE	MAE	*r*^2^	CoD	RMSE	MAE
SSIG	**0.89**	0.89	9619	5424	**0.85**	0.84	13,655	10,040	**0.94**	0.91	9475	5418	**0.85**	0.84	14,654	10,036
Spatial information GP	0.84	0.84	11,414	6865	0.69	0.68	19,507	13,466	0.87	0.83	12,571	8509	0.75	0.72	19,174	13,839
Socio-economic Information GP	0.84	0.84	11,604	6217	0.81	0.80	15,317	11,351	0.94	0.91	9091	5416	0.85	0.82	15,035	10,422

Note: This table presents the results of the ablation study regarding the fivefold validation for per-capita income and median household income estimation at the Tract and the ZIP-level in NYC.

### What predicts income in New York City?

To understand what predicts income in NYC (the spatial feature or the socio-economic feature, or both), data from all districts (with ground truth income data available) were taken as the training set to train our SSIG model.

**Fig. 3:**
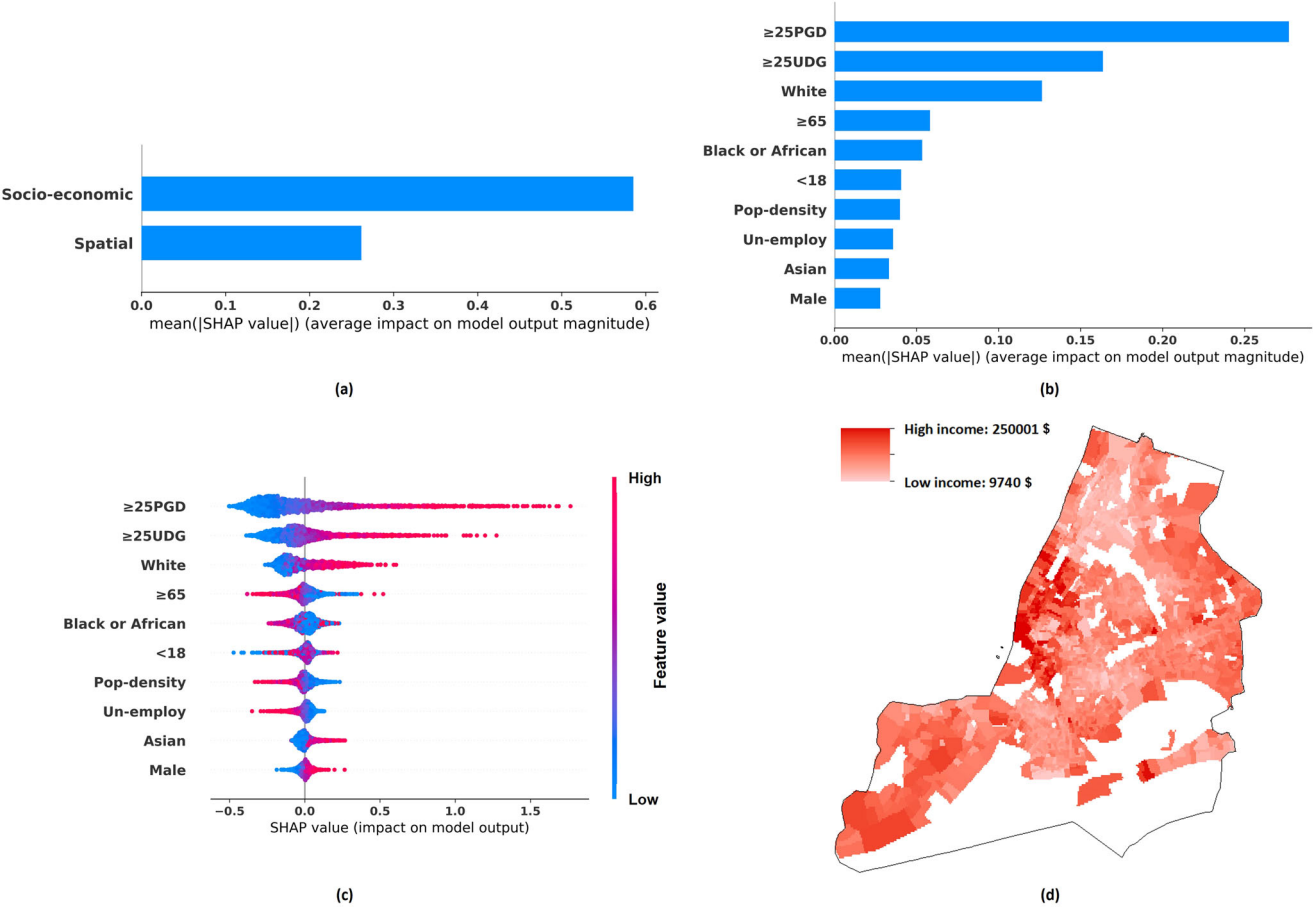
Tract-level median household income distribution and SHAP analysis results. **a** The mean absolute SHAP value indicating the total contribution of spatial or socio-economic features to Tract-level median household income estimation. **b** The mean absolute SHAP value indicating the contribution of individual socio-economic features to Tract-level median household income estimation. **c** The SHAP value indicating the contribution of individual socio-economic features to Tract-level median household income estimation, with each dot corresponding to a value of a particular Tract/district. **d** Tract-level median household income distribution.

**Fig. 4:**
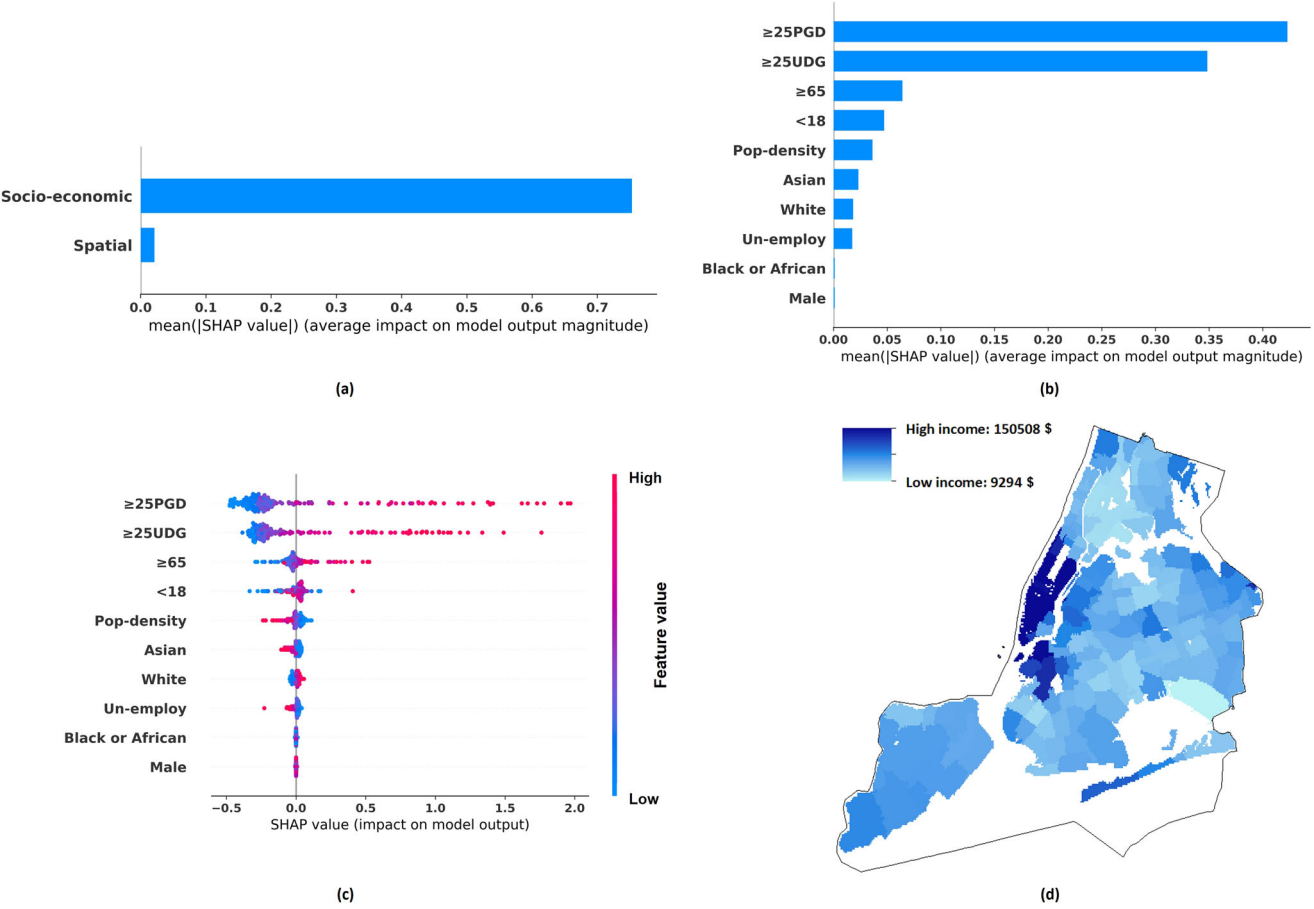
ZIP-level per-capita income distribution and SHAP analysis results. **a** The mean absolute SHAP value indicating the total contribution of spatial or socio-economic features to ZIP-level per-capita income estimation. **b** The mean absolute SHAP value indicating the contribution of individual socio-economic features to ZIP-level per-capita income estimation. **c** The SHAP value indicating the contribution of individual socio-economic features to ZIP-level per-capita income estimation, with each dot corresponding to a value of a particular ZIP region. **d** ZIP-level per-capita income distribution.

**Fig. 5:**
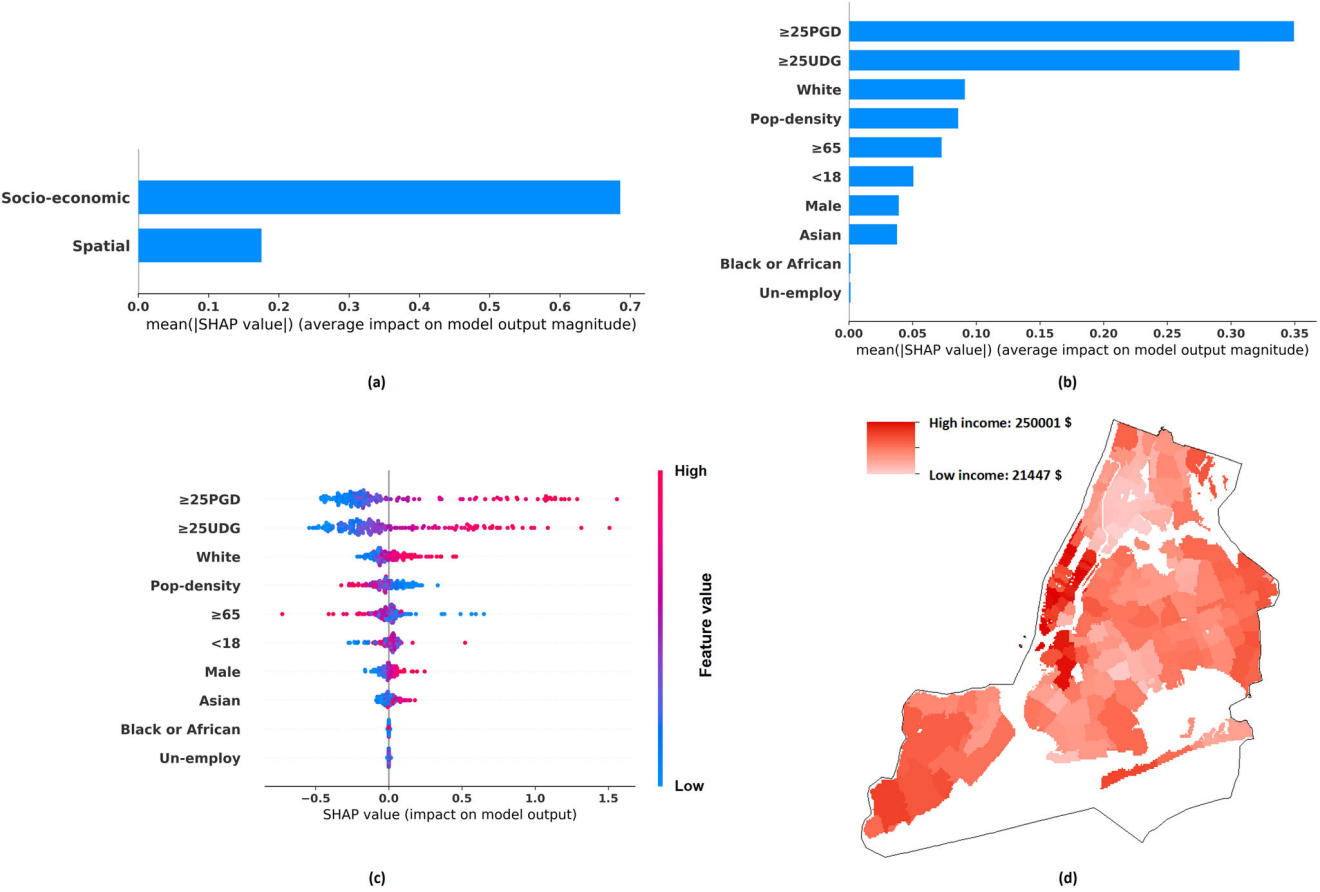
ZIP-level median household income distribution and SHAP analysis results. **a** The mean absolute SHAP value indicating the total contribution of spatial or socio-economic features to ZIP-level median household income estimation. **b** The mean absolute SHAP value indicating the contribution of individual socio-economic features to ZIP-level median household income estimation. **c** The SHAP value indicating the contribution of individual socio-economic features to ZIP-level median household income estimation, with each dot corresponding to a value of a particular ZIP region. **d** ZIP-level median household income distribution.

Figures 2 to 5, and Table 5 show the income distribution and SHAP results, detailing the importance of (a) socio-economic features versus spatial features and (b) different socio-economic features, in estimating per-capita income and median household income of different granularities. The descriptions of socio-economic variables are included in Table 2. Specifically, the total effects of spatial features and socio-economic features are shown in Figs. 2(a), 3(a), 4(a), 5(a). Figures 2(b), 3(b), 4(b), 5(b) illustrate the average absolute value of each socio-economic feature’s SHAP (averaged across all districts), and Figs. 2(c), 3(c), 4(c), 5(c) depict, respectively, the SHAP distribution of each socio-economic feature, with each dot corresponding to a particular district in NYC. The dots are coloured according to the feature’s value, with blue representing a lower value and red representing a higher value. A higher positive SHAP value, such as ≥25PGD, indicates that the corresponding feature would contribute a larger increment in the estimated income values (e.g., see Fig. 2(c)) (Lundberg and Lee, 2017). Figures 2(d), 3(d), 4(d), 5(d) show the corresponding income distributions. Average absolute SHAP values of individual socio-economic variables are presented in Table 5. The table also shows the *p*-value of t-test analysis, which indicates the significance of the difference between the absolute SHAP value of an individual socio-economic indicator for estimating the per-capita income and that for estimating the median household income.

**Table 5:** Average absolute SHAP values of individual socio-economic variables.

Socio-economic variable	Tract-level			ZIP-level		
	Per-capita income	Median household income	*p*-value	Per-capita income	Median household income	*p*-value
**≥65**	**0.0556**	**0.0583**	**0.3638**	**0.0643**	**0.0728**	**0.4354**
**<18**	**0.0595**	**0.0406**	**0.0000**	**0.0472**	**0.0506**	**0.5869**
Male	0.0196	0.0280	0.0000	0.0011	0.0392	0.0000
**≥25UDG**	**0.1895**	**0.1636**	**0.0000**	**0.3484**	**0.3067**	**0.1455**
**≥25PGD**	**0.2619**	**0.2770**	**0.0975**	**0.4230**	**0.3494**	**0.0473**
Un-employ	0.0209	0.0357	0.0000	0.0175	0.0013	0.0000
**Pop-density**	**0.0445**	**0.0400**	**0.0015**	**0.0363**	**0.0858**	**0.0000**
Black or African	0.0210	0.0535	0.0000	0.0011	0.0013	0.3682
Asian	0.0025	0.0332	0.0000	0.0232	0.0378	0.0000
**White**	**0.0723**	**0.1265**	**0.0000**	**0.0183**	**0.0910**	**0.0000**

Note: This table presents the average absolute SHAP value of individual socio-economic variables for the SSIG model used in estimating per-capita income and median household income at the Tract and the ZIP-level in NYC. The variables in BOLD represents a key socio-economic variable in any of the four groups having a SHAP value >0.05.

## Discussions

Two research questions have been put forward in our study. First, by incorporating the values of socio-economic variables of the field surveys into the *Spatial-Information-GP* model, would the income estimation accuracy of high spatial granularity across NYC, a developed economy, be improved? Second, which socio-economic variable(s) in SSIG income estimation model best predict(s) income in NYC?

In general, SSIG achieves outstanding income estimation accuracy compared to the state-of-the-art baseline models (see Table 3). Specifically, SSIG outperforms the baseline models, namely, the *Mixed-Siamese-like-Double-Ridge* model, the *Mixed-Siamese-like-GP* model, the *Mixed-Siamese-like-Random-Forest* model, the *Mixed-Spatial-Siamese-like* model, and the *Mixed-Siamese-like* model, which consist of more complex architectures, or those models with high complexity multi-dimensional data inputs (covering satellite image, street view and house price information). This suggests that these baseline models might take in too much and too complex information as inputs and tend to overfit. It also tends to suggest that the relationship between the socio-economic variables and the income variables collected via the field survey might be more stable, as compared to the relationship between the complex multi-dimensional big proxy data collected via the non-field-survey means, and the income variables collected via the field survey. A more stable relationship can be better generalised for validation, and thus better contribute to the higher fivefold validation accuracy of SSIG. Besides, results of the ablation study indicate that a higher generalisability can be achieved by combining the socio-economic data with spatial information, when developing a GP income estimation model for fine-grained income estimation across a developed context.

Our results have also revealed the relative importance of socio-economic versus spatial contribution to income estimation. Our ablation study shows that in most cases, the GP model based on the socio-economic data only (the *Socio-economic-Information-GP* model) can achieve a higher fivefold validation accuracy, as compared to the counterpart that is entirely based on spatial information (the *Spatial-Information-GP* model). In addition, based on SHAP analysis, Figs. 2(a), 3(a), 4(a), 5(a) have indicated that for high spatial granularity income estimation in NYC, socio-economic variables are more predictive of income as compared to spatial information. As compared to the spatial autocorrelation of income distribution, features capturing socio-economic information across the same district have played a more crucial role when estimating the district-based income level.

Besides, as observed from Fig. 2(b)(c), 3(b)(c), 4(b)(c), 5(b)(c), and Table 5, among the socio-economic variables, ≥25PGD (the proportion of adults ≥25 holding a postgraduate degree) plays the most crucial role in estimating district-based income in NYC, and ≥25UDG (the proportion of adults ≥25 holding a bachelor degree) is the second most crucial factor; a higher ≥25PGD or ≥25UDG contributes to a higher income level in our GP model. This result is consistent with the Mincer equation and the substantive economic literature indicating that an elevation in the educational level can increase the chance of higher salaries, based on the premise that a higher level of educational attainment enhances productivity (Becker, 2009; Dodge, 2003; Fullerton Jr et al., 2014; Fullerton Jr et al., 2010; Fullerton, 2001; Gottlieb and Fogarty, 2003; Jones, 2001; Patrinos, 2016; Psacharopoulos and Patrinos, 2004; Rauch, 1993; Rosenzweig, 1995; Simon, 1998; Welch, 1970).

In terms of Race and Sex, our results show that higher White (the proportion of the White population) is associated with a higher income level in NYC, and higher Male (the proportion of Male persons) is associated with a higher median household income level in NYC. These findings support the existence of race and sex inequality across the urban region in developed economies (Akee et al., 2019; Bailey et al., 2014; Hamilton, 1973; Reardon et al., 2015). In addition, the contribution of White to income estimation is relatively higher than Male. This indicates that the income gap is more attributable by Race. When recruiting candidates of higher-paid jobs, Race might be a more important consideration than Sex. Though Race or Sex inequality during the recruitment process was mentioned, further verification is needed (Baert, 2018; McCarthy and Cheng, 2018; Skaggs and Bridges, 2013). In addition, Race has been considered carrying a stronger effect on annual income increment than Sex. Liu et al. (2019) indicated that as the number of work years increases, the gap of income increment attributable by Race was significantly larger than that by Sex.

In general, Pop-density (population density) and Un-employ (unemployment rate) are negatively related to the district-based income, while their contributions to the income levels in NYC are moderate. Negative correlation can be found in Figs. 2(c), 3(c), 4(c), and 5(c). Specifically, the red dots in these figures represent the samples that exhibit the higher values of corresponding variables, as most red dots corresponding to Pop-density and Un-employ are distributed at the left with negative SHAP values, indicating higher variable values can contribute to lower estimated incomes. The negative correlation between Pop-density and Income at the district-level in NYC can be explained by the fact that the more populated districts may risk a higher chance of traffic congestion and pollution (Chang et al., 2021; Eriksson and Zehaie, 2005; Fullerton Jr et al., 2014). These negative factors may aggravate the local economy, while citizens of a higher income level may try to avoid residing in these districts (Finkelstein et al., 2003). The effect of Un-Employ on income estimation has been consistent with the general expectation that a high unemployment rate can deteriorate the average district-based income (Acs, 2008).

For Age, the impacts of both <18 (the proportion of the population who are younger than 18) and ≥65 (the proportion of the population who are 65 or above) on different types of income and of different granularities are ambiguous/inconsistent. Although most children or young people smaller than 18 do not work or hold a full-time job, their parents or family members may still enjoy an income level way above the average (Fullerton Jr et al., 2014). As for the population ≥65, though retirement may imply a reduction in income level for a certain part of the population, it is also possible that some other parts of the population may still experience an income rise due to an increase in welfare payment or pension (Fullerton Jr et al., 2014; Fullerton, 2001).

We have also compared same socio-economic variables’ average absolute SHAP values across different models (see Table 5). Although previous studies investigated the effects of socio-economic variables on fine-grained district-based income estimation in developed contexts, their relative effects on the same variable of per-capita income and median household income have yet to be fully explored (Almada, 2004; Fullerton Jr et al., 2014; Fullerton Jr et al., 2010; Fullerton, 2001; Morales, 2012). Since the SSIG model’s inputs and outputs have been normalised before model training, the SHAP results are comparable. To reduce the effect of randomness, we focus on the variables that have achieved consistent SHAP results across two different models estimating income at both the ZIP and the Tract-level, with the difference of the absolute SHAP values ≥0.01, and the difference is statistically significant at least the ZIP-level or the Tract-level (*p* < 0.05). To ensure that we focus on variables that are strongly attributable to income, a variable in any of the four columns in Table 5 having an absolute SHAP value ≥0.05 is taken as key variable influencing income (the estimated per-capita income or the estimated median-household income at the Tract-level or the ZIP-level, or at both levels). Three socio-economic variables, including ≥25UDG, ≥ 25PGD, and White, have been selected based on the above criteria. Specifically, the estimated per-capita income is more sensitive to ≥25UDG (the proportion of adults ≥25 holding a bachelor degree) than the estimated median household income. A possible explanation being that holders of a bachelor degree might concentrate in the high-income level household, instead of the median-income level one, which can be partly explained by one’s tendency to marry someone else of the same educational level (Domingue et al., 2014; Eika et al., 2019; Esteve et al., 2012; Hou and Myles, 2008). One may more likely have a bachelor degree, if the other party of the household has obtained a bachelor degree also; in such cases, their combined household income would generally be higher than the median income at the district level (a household consisting of a couple is generally more capable of earning a higher income than a single member household). Though there might be arguments supporting the view that a man might marry a woman of a lower educational level, this observation may not stand, as the gender gap in education has increasingly been shrinking, with more women holding higher educational degrees (Esteve et al., 2012). Furthermore, the results showing that the estimated per-capita income having a higher sensitivity to ≥25PGD at the ZIP-level implies that findings at the ZIP-level might be more credible than that at the Tract-level, given that the input data are of a higher quality. Given that our analysis mentioned above on ≥25UDG should be applicable to ≥25PGD, and the SHAP comparison result of ≥25PGD at the ZIP-level (instead of the Tract-level) is consistent with that of ≥25UDG, our SHAP results at the ZIP-level should be more credible than that at the Tract-level. Finally, the estimated median household income is more sensitive to White (the proportion of the White population), as compared to the estimated per-capita income. For districts having a higher White population, in the median household income group, the proportion of White would be higher than the White proportion at the district level; for districts of a lower White population, opposite result is observed; this implies that district-based median household income might be more sensitive to the White as compared to average per-capita income. This might be due to one’s higher propensity to marry partners of the same race when one is living in a racially homogeneous region (Bécares et al., 2009; Borrell et al., 2021; Fu, 2000; Fu et al., 2001; White and Borrell, 2011). Some might argue inter-racial marriage has been increasingly popular over the recent years (Borrell et al., 2021). However, inter-racial marriage is more commonly observed in a racially heterogeneous region (Borrell et al., 2021). Individuals living in these regions tend to marry someone of a different race, when racial discriminations in these regions is not strong and strong social support to support such couples can be found (Bécares et al., 2009; Borrell et al., 2021; White and Borrell, 2011). However, in racially homogeneous regions, people, e.g., the White, may be relatively resistant to inter-racial marriage, given the relatively weak social support (Borrell et al., 2021). However, questions might arise as why marrying someone with the same educational level and race can give contradictory results, as shown by SHAP results. As compared to the estimated per-capita income, the estimated median household income is less sensitive to ≥25UDG, but more sensitive to White. A possible reason being that the income enhancement effect of the increase in the proportion of ≥25UDG is much stronger than that of the increase in the proportion of the White population, as shown in Figs. 2(c), 3(c), 4(c), and 5(c). Hence, for a household consisting of a couple both having a bachelor degree or above, a higher income above the median level is expected. However, for a household consisting of only White family members, a median-income might still be possible.

Overall, our results have implied that, though income inequality due to Sex and Race are still prevalent in NYC, a higher educational attainment (holding a bachelor or higher degree) can potentially rebalance the income distribution and reduce income inequality. Local decision-makers of these developed economies are thus encouraged to provide higher education opportunities to citizens residing in the low-income districts, thus reducing the potential social conflicts triggered by urban income inequality, thereby improving urban sustainability (Ebrahimi et al., 2022; Malin et al., 2020; Schneider, 2016). Household selection can be an additional dimension for understanding the distribution of district-based inequality. In particular, people tend to live in districts where neighbours are sharing similar socio-economic characteristics (e.g., those sharing similar race or educational level). This may aggravate between-district inequality and other socio-economic-driven inequalities. Such phenomenon can influence policy decisions, since moving people to different districts can effectively lessen district-based inequality, but may potentially create public discontents and new social problems. Policy analyses conducted using correlational studies should be interpreted with caution. For instance, household selection might present challenges for causality-driven socio-economic analyses (e.g., race, educational level). The SHAP analysis deployed for this study should be taken as correlational instead.

This paper has only examined the socio-economic variables that influence district-based income in NYC during 2015–2019, without covering 2020 or beyond, the pandemic period, when SARS-CoV-2 infection and mortality have started to change the income landscape. Dang and Nguyen (2021) indicated that women were more likely to permanently lose their jobs due to the pandemic, and Abedi et al. (2021) suggested that African Americans were more vulnerable during the pandemic. This might intensify intra-city income inequality. Besides, Qian and Fan (2020) showed that people of a higher educational attainment will have a lower chance of income loss during the period. This observation strengthens our view that providing more education opportunities to the lower-income districts in NYC can potentially reduce the income gap and hence between-district inequality. Future studies can further investigate the interaction between SARS-CoV-2 and income inequality, while adapting our district-based income estimation models to cater for the pandemic context.

Our study serves as a valuable reference for future studies that explore between-district income inequality in other cities. Even though between-district income inequality studies have been explored in many different parts of the world, exact Gini indexes might not be directly comparable with those in Table 1. For instance, Kataoka showed that Between-district Gini Index for per-capita income in Indonesia ranged from 0.1 to 0.15 during 2004 to 2018 (Kataoka, 2022). Between-district Gini Index ranged from around 0.15 to 0.25 in Odisha (a state in India) during 1995 to 2011 (Mahakur and Nayak, 2019). Since the size of the districts in these countries/cities is different from that in NYC, cross-comparison of these cities at different developmental stages is restricted. Besides, Between-district Gini Index for developed cities calculated based on high granularity district-based income distribution is yet to be developed (Almada, 2004; Fullerton Jr et al., 2014; Fullerton Jr et al., 2010; Fullerton, 2001; Morales, 2012), and our study in NYC fills the gap.

## Conclusion

Our study has presented the key novelties in model development and interpretation, and novel findings yet to be thoroughly investigated in the previous fine-grained district-based income estimation studies covering the developed economies (Almada, 2004; Fullerton Jr et al., 2014; Fullerton Jr et al., 2010; Fullerton, 2001; Morales, 2012). We have developed a novel SSIG model by taking both socio-economic variables and spatial information as the input features to a GP model. We have also calculated the SHAP values of individual socio-economic variables to evaluate their corresponding feature impacts (Lundberg and Lee, 2017), and their relative contributions to income. The results show that the SSIG model could achieve outstanding estimation accuracy for per-capita income and median household income at the Tract-level and the ZIP-level in NYC. The better performance of SSIG, as compared to other GP models based entirely on socio-economic information or spatial information, has indicated that the combination of socio-economic and spatial information can contribute to higher income estimation accuracy. In addition, by incorporating inputs gathered from both field surveys and machine-learning big data-based techniques, SSIG outperforms other baseline models. This implies that, instead of restricting on either machine-learning big data-based inputs, or field survey-based statistical analyses, future income estimation studies can capitalise on the best of both worlds. The SHAP results have indicated that ≥25PGD and ≥25UDG, the two higher educational attainment variables, are most critical in determining district-based income in NYC, with White and Male contributing to income inequality. Our study has also observed that the income gap is more associated with Race than Sex. We have proposed that the stronger effect of Race on the Income gap as compared to Sex, might be attributable to its stronger effect on recruitment procedure and annual income increment (Baert, 2018; Liu et al., 2019; McCarthy and Cheng, 2018; Skaggs and Bridges, 2013). Policymakers are encouraged to pay more attention to any inherent socio-economic obstacles to achieving greater urban sustainability, including sex and racial-driven income inequality. They can narrow the income divide in the developed economy of NYC by promoting higher educational attainment for residents of the lower-income districts. Besides, our results indicate that the estimated per-capita income is more sensitive to ≥25UDG as compared to the estimated median household income. A possible explanation being that people holding a bachelor degree might concentrate in households of top income level, instead of households of median income level. Such an assumption can be supported by people’s tendency to marry someone of the same educational level (Domingue et al., 2014; Eika et al., 2019; Esteve et al., 2012; Hou and Myles, 2008). The results on ≥25PGD have indicated that statistical results at the ZIP-level might be more credible than that at the Tract-level due to the higher data quality. In addition, the estimated median household income is more sensitive to White. When compared to the proportion of White population in a district, for districts of a higher White population, households having a median-level income have a higher the proportion of White people, whilst in districts of a lower White population, the situation is reversed. Such phenomenon might be attributable to one’s higher chance or incentive to marry a partner of the same race, when one is living in a district where many of these people are sharing the same race as that person (Bécares et al., 2009; Borrell et al., 2021; Fu, 2000; Fu et al., 2001; White and Borrell, 2011). In addition, our work has illustrated that SHAP can be used in future machine-learning-based socio-economic study to investigate the relative contribution of socio-economic variables on a certain predicted outcome, and in this case, between-district inequality. While the empirical study itself has reinforced former studies that having a bachelor or a postgraduate degree are the two most important predictors of income across a developed context, our work goes beyond to illustrate that the relative contributions of socio-economic variables can be visualised and determined via SHAP analysis, whenever machine-learning big data-based techniques are deployed for future socio-economic analyses.

While our study has made significant contributions to methodology and findings for interpretative machine-learning big data-based socio-economic analysis, we humbly acknowledge the limitations of our study. First, given that American Community Survey has conducted less frequent field surveys on smaller districts, our results based on small and less frequently collected samples should be taken with a grain of salt. Second, given that the Tract/ZIP-level income data from American Community Survey are based on a 5-year average instead of an annual average, the values of Between-district Gini Index/annual estimated income should be taken with caution. In addition, future studies can investigate further the interaction effects of different socio-economic variables on per-capita income or median household income estimation based on the SHAP interaction values, whenever such analyses are allowed (Lundberg et al., 2020). This can be made possible if SHAP interaction effect calculation is permissible with new software that cater for a wider variety of machine-learning models.

## Supplementary information

Supplementary Information

## Data Availability

The data generated in this study and code can be made available upon request to the corresponding authors.
