# Supplementary material for: What dictates income in New York City? SHAP analysis of income estimation based on Socio-economic and Spatial Information Gaussian Processes (SSIG)
Source: Humanit Soc Sci Commun. 2023 Feb 15;10(1):60. doi: 10.1057/s41599-023-01548-7 (PMC9930030; doi:10.1057/s41599-023-01548-7)
Supplement: Supplementary file 1 — Supplementary Information [file 41599_2023_1548_MOESM1_ESM.docx]

**What dictates income in NYC? SHAP analysis of income estimation based on** **Socio-economic and Spatial Information Gaussian Processes (SSIG)**

**Supplementary Information**

**Ruiqiao Bai^1^, Jacqueline C. K. Lam^1, *^, Victor O. K. Li^1, *^**

^1^Department of Electrical and Electronic Engineering, The University of Hong Kong, China
^*^Corresponding authors ([jcklam@eee.hku.hk](mailto:jcklam@eee.hku.hk), vli@eee.hku.hk)

**Table S1. A review of socio-economic indicators used for estimating fine-grained district-based income in developed economies**

| **Category** | **Indicator** | **Reference** |
| --- | --- | --- |
| **Education** | Percentage of adults 25 and over who dropped out of high school | (Fullerton, 2001; Almada, 2004) |
|  | Percentage of adults 25 and over who graduated from high school | (Almada, 2004; Fullerton Jr et al., 2010; Morales, 2012; Fullerton Jr et al., 2014) |
|  | Percentage of adults 25 and over who attended college | (Fullerton, 2001; Almada, 2004; Fullerton Jr et al., 2010; Morales, 2012; Fullerton Jr et al., 2014) |
|  | Percentage of adults 25 and over who graduated from college | (Fullerton, 2001; Almada, 2004) |
|  | Percentage of adults 25 and over holding (or at least with) one bachelor degree | (Fullerton Jr et al., 2010; Morales, 2012; Fullerton Jr et al., 2014) |
|  | Percentage of adults 25 and over holding postgraduate degrees | (Fullerton Jr et al., 2010) |
|  | Percentage of high school graduates who intend to attend a two or four-year college | (Dodge, 2003) |
|  | Total battery Indiana State Test for Economic Progress score (a measurement of student academic performance) | (Dodge, 2003) |
| **Employment** | Female labor force participation rate | (Almada, 2004; Fullerton Jr et al., 2014) |
|  | Unemployment rate | (Dodge, 2003) |
|  | Ratio of manufacturing jobs | (Dodge, 2003) |
| **Age** | Percentage of population who are 65 and above | (Fullerton, 2001; Almada, 2004; Morales, 2012; Fullerton Jr et al., 2014) |
|  | Percentage of population who are 18 and younger | (Fullerton, 2001; Almada, 2004; Morales, 2012; Fullerton Jr et al., 2014) |
| **Population** | Number of persons per area | (Fullerton Jr et al., 2010; Morales, 2012; Fullerton Jr et al., 2014) |
|  | Whether the population exceeds a threshold (e.g., 599999) | (Fullerton, 2001; Almada, 2004) |
| **Infrastructure** | Whether a commercial airport is in the region | (Fullerton Jr et al., 2010) |
|  | Commercial airport takeoffs and landings per capita | (Morales, 2012; Fullerton Jr et al., 2014) |
|  | Distance to the nearest commercial airport | (Fullerton Jr et al., 2010) |
|  | Whether interstate highway traverses the region | (Fullerton Jr et al., 2010) |
|  | Length of highways other than the interstate highway | (Fullerton Jr et al., 2010) |
|  | Centerline highway length per capita owned by the state | (Morales, 2012; Fullerton Jr et al., 2014) |
|  | Whether the region locates in a metropolitan statistical area | (Dodge, 2003) |
| **Private capital stock** | Per-capita private sector property tax valuation | (Morales, 2012; Fullerton Jr et al., 2014) |
| **Language proficiency** | Percentage of monolingual English households | (Fullerton, 2001) |
|  | Percentage of bilingual households | (Fullerton, 2001) |
|  | Percentage of monolingual Spanish households | (Fullerton, 2001) |
|  | Percentage of people that speaks English only | (Almada, 2004; Morales, 2012; Fullerton Jr et al., 2014) |
|  | Percentage of people who speaks English and Spanish | (Almada, 2004) |
|  | Percentage of people who speaks Spanish only | (Almada, 2004) |
| **Border proximity** | Whether the region is along the border with Mexico | (Fullerton, 2001; Almada, 2004) |

Note: The table presents socio-economic indicators used in previous field survey-based studies for fine-grained district-based income estimation across developed economies.

**Table S2. Data source and time frame**

| **Indicator/Data** | **Data source** | **Time frame** |
| --- | --- | --- |
| Per-capita income | American Community Survey (a five-year estimate) data provided by Census Reporter | 2015-2019 |
| Median household income | American Community Survey (a five-year estimate) data provided by Census Reporter |  |
| ≥25UDG | American Community Survey (a five-year estimate) data provided by Census Reporter |  |
| ≥25PGD | American Community Survey (a five-year estimate) data provided by Census Reporter |  |
| Un-employ | American Community Survey (a five-year estimate) data provided by Census Reporter |  |
| ≥65 | American Community Survey (a five-year estimate) data provided by Census Reporter |  |
| <18 | American Community Survey (a five-year estimate) data provided by Census Reporter |  |
| Male | American Community Survey (a five-year estimate) data provided by Census Reporter |  |
| Black or African | American Community Survey (a five-year estimate) data provided by Census Reporter |  |
| Asian | American Community Survey (a five-year estimate) data provided by Census Reporter |  |
| White | American Community Survey (a five-year estimate) data provided by Census Reporter |  |
| Total population of district | American Community Survey (a five-year estimate) data provided by Census Reporter |  |
| Area size of district | Boundary shapefile provided by Census Reporter |  |
| Latitude and longitude of district centroid | Boundary shapefile provided by Census Reporter |  |
| House price | NYC Department of Finance | 2019 |
| Latitude and the longitude of each building | NYCityMap | 2021 |
| Daytime satellite image | NYC then and now | 2018 |
| Street view | Google Street View Static API | 2018-2020 |

Note: The table presents the source and time frame of data used in this study.
